# Performance perception of Canadian dairy producers when transitioning to an automatic milking system

**DOI:** 10.3168/jds.2021-0082

**Published:** 2021-06-23

**Authors:** M. Duplessis, E. Vasseur, J. Ferland, E.A. Pajor, T.J. DeVries, D. Pellerin

**Affiliations:** 1 Centre de recherche et développement de Sherbrooke, Sherbrooke, QC, J1M 0C8, Canada; 2 Department of Animal Science, McGill University, Sainte-Anne-de-Bellevue, QC, H9X 3V9, Canada; 3 Département des sciences animales, Université Laval, Québec, QC, G1V 0A6, Canada; 4 Department of Production Animal Health, University of Calgary, Calgary, AB, T2N 1N4, Canada; 5 Department of Animal Biosciences, University of Guelph, Guelph, ON, N1G 2W1, Canada

## Abstract

Adoption of automated milking systems (AMS) has increased exponentially around the world in recent years. The objective of this observational study was to evaluate how producer perception of changes in cow-average milk yield and somatic cell count (SCC) compared with the actual changes in their herds after the introduction of AMS in Canadian commercial dairy herds. Data were collected (in 2014 and 2015) through a survey of 97 Canadian dairy herds that shifted to AMS from 2000 to 2014. Producers were asked their perception about milk yield and SCC changes (increase, decrease, or no change) after AMS introduction. Actual herd performance data were obtained from dairy herd improvement organizations. Differences between the 12-mo rolling herd-average milk yield (kg/cow per year) and SCC (cells/mL) at the closest test 2 yr after transitioning to AMS and at the last test before the transition were calculated and compared with the producer perception answers. After AMS adoption, milking herd size, milk yield, SCC, and number of AMS units per herd averaged (± standard deviation) 99.8 ± 54.4 cows, 9,619 ± 1,354 kg/cow per year, 248,825 ± 97,286 cells/mL, and 1.9 ± 1.1 units, respectively. On average, after AMS introduction, herd size, milk yield, and culling rate increased by 11.3 cows, 441 kg/cow per year, and 1.3%, respectively, and calving interval decreased by 7 d. For producers who perceived an increase, actual milk yield and SCC increases averaged (mean ± standard deviation) +534 ± 1,003 kg/cow per year and +56,679 ± 66,662 cells/mL, respectively. Alternatively, for producers who perceived a decrease, actual milk yield and SCC decreases averaged −984 ± 658 kg/cow per year and −26,976 ± 94,099 cells/mL, respectively. An actual milk yield change of +83.1 ± 1,113.3 kg/cow per year and an SCC change of +6,135 ± 72,609 cells/mL were observed in the herds in which the dairy producers perceived no change with the AMS introduction. Hence, dairy producers were, on average, able to discern their actual milk yield and SCC changes after AMS adoption. However, the proportions of dairy producers who accurately perceived their actual milk yield and SCC changes after AMS introduction were 39.4% for milk yield (increase: 36.3%; decrease: 100.0%; and no change: 45.5%) and 46.7% for SCC (increase: 50.0%; decrease: 39.0%; and no change: 54.1%). From these results, we concluded that several dairy producers distorted their actual milk yield and SCC changes or were not fully aware of those changes.

## Highlights

• This study focused on producer perception after adoption of an automated milking system.
• A total of 39.4% of dairy producers accurately perceived their actual milk yield changes, and 46.7% of dairy producers accurately perceived their actual SCC changes.
• A number of dairy producers distorted their actual performance changes.

## Graphical Abstract

**Figure:**
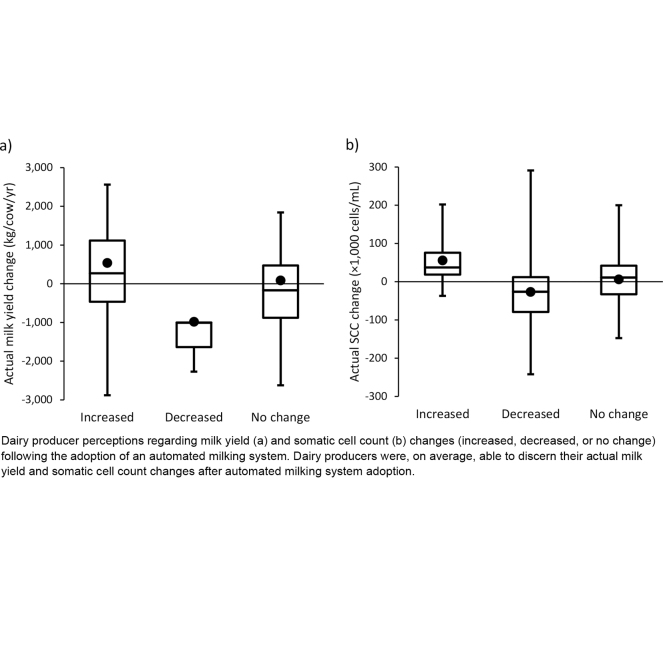
**Summary:** A survey was conducted to evaluate the ability of dairy producers to discern changes in cow-average milk yield and somatic cell count (SCC) when transitioning to an automatic milking system. On average, dairy producers were able to discern actual milk yield and SCC changes. Nevertheless, 64% of the respondents perceived an increase in milk yield and 61% perceived a decrease in SCC after the milking system change, whereas it was not the case in reality. These results indicate that some dairy producers either distorted their actual milk yield and SCC changes or were not aware of those changes.

The first automated milking system (**AMS**) was introduced in 1992 on a commercial farm located in the Netherlands (Steeneveld et al., 2012). Since then, the number of dairy farms equipped with an AMS has exponentially increased worldwide. In 2019, 12% of Canadian Holstein dairy farms participating in DHI recording operated an AMS (Canadian Dairy Information Centre, 2020). Two-thirds of dairy producers who have transitioned to AMS mentioned social and family relationships as the primary reason for adopting the technology (Mathijs, 2004). Flexible working hours are often cited by dairy producers as the biggest AMS advantage (Hansen, 2015). In a survey of 217 Canadian AMS dairy producers, the vast majority of them stated that the transition to AMS improved their quality of life and that they would recommend AMS to other producers (Tse et al., 2018b).

Another advantage of transitioning to AMS is the potential improvement in milk production. In a survey of Spanish dairy producers, 58% of producers mentioned that one of the reasons for installing an AMS was the expected increase in milk yield (Castro et al., 2015). A review on AMS reported that a 2 to 12% increase in milk yield has been observed for cows that transitioned from 2 to >2 milkings per day (Jacobs and Siegford, 2012). Special attention should be given to udder health, which has been reported in several studies to deteriorate within the first year of AMS introduction (Hovinen and Pyörälä, 2011). A survey conducted by Tse et al. (2018a) revealed that Canadian producers perceived, on average, greater milk yield and no change in milk quality (SCC) following AMS adoption. To our knowledge, no study has compared the actual change in milk yield and quality following AMS introduction with the producer perception of that change. Hence, this study was undertaken to evaluate how dairy producer perception of changes in cow-average milk yield and SCC compared with the actual changes that occurred in their herds after the introduction of AMS in Canadian commercial dairy herds.

Data used in this study were collected as part of a larger study aimed at evaluating producer experience when transitioning to AMS (Tse et al., 2017, 2018a,b). Before contacting participants, institutional human ethics certification was obtained (University of Calgary, certification no. REB14-0149_MOD1). Briefly, producer contact names (n = 530) were provided through an AMS dealer client list in Canada (Lely Canada and DeLaval Canada), Alberta Milk, and the Dairy Farmers of Manitoba. To participate in the study, the main breed of the herd had to be Holstein. All producers on the list were contacted, and willingness to participate in the survey study was confirmed. Overall, 217 Canadian dairy producers participated in the study (Tse et al., 2017, 2018a,b). For the current study, milk recording information was required; that information was missing for 120 herds following AMS adoption, and thus survey data from those herds were not used. Hence, from May 2014 to February 2015, data from 97 Canadian dairy herds [British Columbia, n = 2; Alberta, n = 19; Saskatchewan, n = 1; Manitoba, n = 7; Ontario, n = 39; Québec, n = 27; and Atlantic provinces (New Brunswick, Nova Scotia, Prince Edward Island), n = 2] that had shifted to AMS from 2000 to 2014 were used. As part of the larger study, producers were contacted by phone, by email, or in person and asked to complete a 2-part survey (as described by Tse et al., 2017). Producers were aware that they could end their participation in the study at their request. Producers were asked for their perception on how cow-average milk yield and SCC changed after AMS introduction (increase, decrease, or no change). No threshold on what could be considered an increase, decrease, or no change in performance was provided to the producers. The survey also included questions related to the number of cows before and after transitioning to AMS and other descriptive information. Milk production and management data were provided by the Canadian DHI organizations [Lactanet (formerly Valacta and CanWest DHI)]. Data from DHI milk tests were used to compare producer perception of milk yield and SCC changes with the actual milk yield and SCC changes after the transition to AMS compared with before the transition. Differences between the 12-mo rolling herd-average milk yield (kg/cow per year) and SCC (cells/mL) at the closest milk test 2 yr after transitioning to AMS (mean ± SD; 0.66 ± 1.45 mo relative to the 2 yr after the transition) and at the last test before the transition (0.67 ± 1.33 mo) were calculated. The 12-mo rolling herd average included animal performance during the 365-d period before the last DHI test date. This methodology allowed cows to be fully adapted to the AMS before considering their milk performance and to overcome any effects that may have been caused by barn construction or other changes that occurred in the period immediately before AMS introduction. The performance difference was then classified as an actual increase, decrease, or no change. To determine what was considered an actual increased performance, a 1-sided TTEST procedure was performed in SAS (version 9.4; SAS Institute, 2012) with positive performance differences between after the transition and before the transition to obtain the 95% confidence interval, for which a performance difference could be considered different from 0. The same was done with negative performance differences. The lower and upper 95% confidence interval results, when using the respective positive and negative differences, were used as thresholds to determine whether producer perceptions were in line with reality. Data inside the 2 thresholds could not be considered different from 0 and thus were classified as stable performance (no change) following AMS adoption. Performance indicators before and after the introduction of AMS, such as herd milk yield and SCC averages and culling rate, were determined to distinguish herds in which producer perception and actual results were the same from those in which they were different.

Descriptive statistics were obtained using the MEANS procedure of SAS. Comparisons between actual performance and producer perception within categories (increase, decrease, or no change) were analyzed with a nonparametric Kruskal-Wallis test (chi-squared approximation) using the NPAR1WAY procedure of SAS, as the normality assumption was violated according to visual assessment of the residuals. Performance indicators were compared among perception categories (perceived change or not) using the TTEST procedure of SAS. Equality of variances was assessed; when they were unequal, *P*-values were obtained using the COCHRAN test. Results were considered significant when *P* ≤ 0.05 and a tendency when 0.05 < *P* ≤ 0.10. Valid data for both perception and reality were obtained for 94 and 92 herds for cow-average milk yield and SCC, respectively.

Descriptive data for before and after AMS transition are presented in Table 1. Before AMS introduction, average herd size was 88.5 cows, producing on average 9,178 kg/cow per year. Following AMS adoption, herd size, milk yield, and culling rate increased on average by 11.3 cows, 441 kg/cow per year, and 1.3%, respectively, and calving interval decreased by 7 d. Bugueiro et al. (2019) reported similar results regarding the increase in milk yield and culling rate following AMS transition in Spanish herds. Number of cows per AMS unit averaged 54.4 cows, which is similar to that reported by Castro et al. (2012) and less than the typically described AMS capacity of 60 milking cows (Rotz et al., 2003). In 2014, the typical Canadian dairy farm had 77 cows producing 8,984 kg/yr, and the calving interval and culling rate averaged 424 d and 39.3%, respectively (Valacta, 2015). Results from the current study demonstrate that herds shifting to AMS were characterized by higher producing cows and greater herd size compared with the typical Canadian dairy farm at that time. The number of participating dairy herds within each province in our study closely reflected the actual national distribution of dairy herds; therefore, we performed a single global analysis for all herds across Canada.

**Table 1:** Descriptive statistics of the 97 participating herds that adopted automated milking systems (AMS)

Item^1^	Average	SD	Minimum	Maximum
Average cows before AMS (no.)	88.5	52.5	32	420
Average cows after AMS (no.)	99.8	54.4	38	340
Milk yield before AMS (kg/cow per year)	9,178	1,149	5,857	12,109
Milk yield after AMS (kg/cow per year)	9,619	1,354	5,357	12,049
SCC before AMS (cells/mL)	248,995	94,646	89,000	625,000
SCC after AMS (cells/mL)	248,825	97,286	86,000	642,000
Calving interval before AMS (d)	427	32	383	572
Calving interval after AMS (d)	420	28	379	514
Culling rate before AMS (%)	38.5	11.9	12.0	82.1
Culling rate after AMS (%)	39.8	11.1	16.1	86.1
AMS units per farm^2^ (no.)	1.9	1.1	1	9
Cows per AMS unit^2^, ^3^ (no.)	51.6	19.2	35	75

1 For all items except AMS units per farm and cows per AMS unit, data are from the Canadian DHI organizations. Data from after AMS transition were obtained from the closest milk test 2 yr after transitioning to AMS (mean ± SD; 0.66 ± 1.45 mo relative to the 2 yr after the transition), and data from before AMS transition were obtained from the last test (0.67 ± 1.33 mo) before the transition.

2 Information was obtained from a survey of the producers.

3 Data are based on 94 herds.

The Kruskal-Wallis test revealed that data in each perception category (increase, decrease, or no change) were not from the same distribution, meaning that the actual changes were different within the categories (*P* < 0.03; Figure 1). Out of 94 herds, 80 producers perceived a milk yield increase, 3 perceived a decrease, and 11 perceived no change following AMS transition. Out of 92 herds, 14 producers perceived an SCC increase, 41 perceived a decrease, and 37 perceived no change after AMS adoption. For those who perceived an increase, increases in milk yield and SCC actually averaged (mean ± SD) +534.1 ± 1,002.7 kg/cow per year and +56,679 ± 66,662 cells/mL, respectively, whereas for those who perceived a decrease, milk yield and SCC decreases actually averaged −983.7 ± 657.6 kg/cow per year and −26,976 ± 94,099 cells/mL, respectively (Figure 1). An actual milk yield change of +83.1 ± 1,113.3 kg/cow per year and an SCC change of +6,135 ± 72,609 cells/mL were observed in the herds in which the dairy producers perceived no change with AMS introduction. Thus, dairy producers were, on average, able to discern milk yield and SCC changes after AMS introduction. This may be due to the fact that these 2 production variables are widely available through monthly DHI reports as well as milk payments (based on samples taken at every bulk tank pick up). On the contrary, Vasseur et al. (2012) and Higginson-Cutler et al. (2015) reported low Canadian producer ability to estimate actual juvenile calf mortality and cow lameness, which are much less documented than milk yield and SCC.

**Figure 1:**
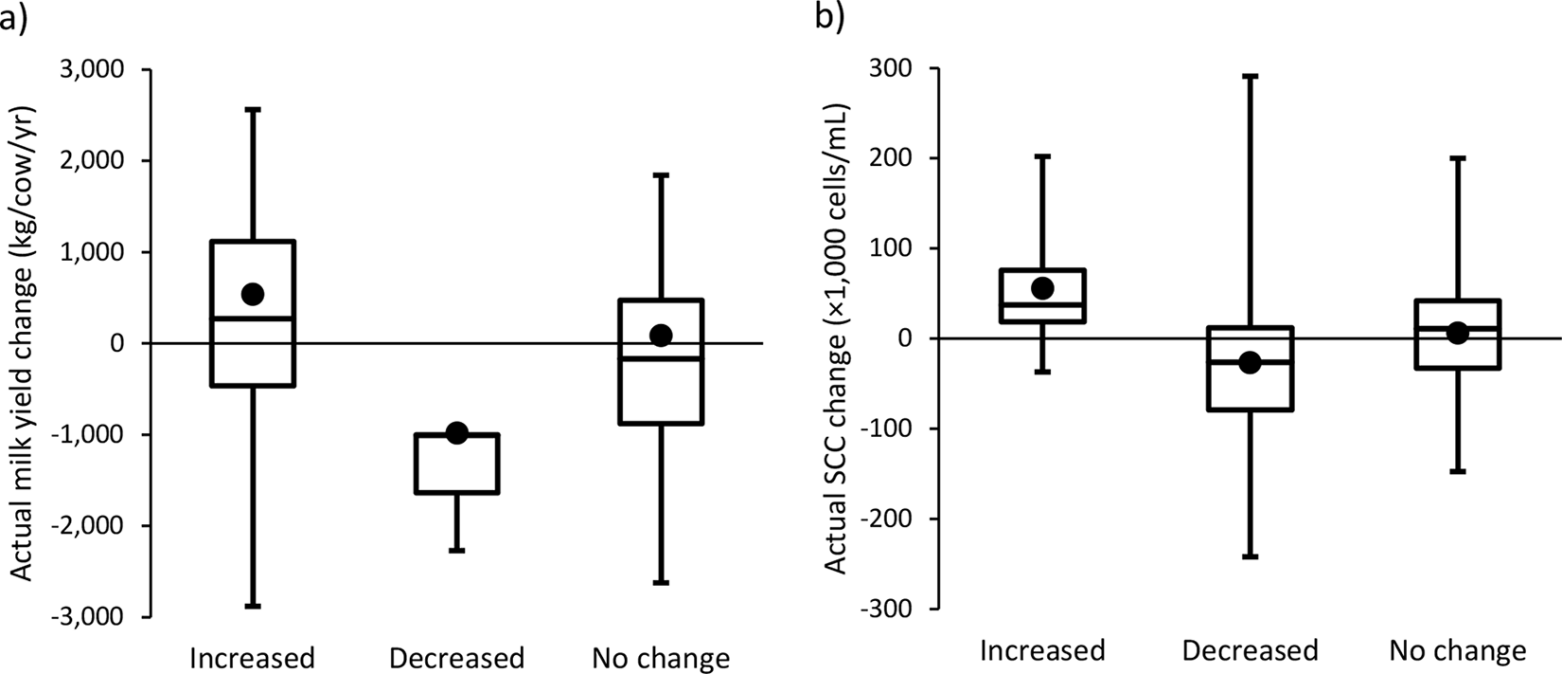
Box plots representing dairy producer perceptions regarding (a) milk yield and (b) SCC changes (×10^3^ cells/mL; increase, decrease, or no change) associated with the actual change following automated milking system adoption. Actual changes were different within the categories (*P* < 0.03). The black circles represent average actual changes, whiskers represent minimum and maximum values, the midline is the median, and the box represents 25 and 75th quartiles. Valid data for both perception and reality were obtained for 94 and 92 herds for milk yield (perception: increased, n = 80; decreased, n = 3; no change, n = 11) and SCC (perception: increased, n = 14; decreased, n = 41; no change, n = 37), respectively. Milk yield and SCC changes were calculated as differences between the 12-mo rolling herd-average milk yield and SCC at the closest milk test 2 yr after transitioning to an automated milking system (mean ± SD; 0.66 ± 1.45 mo relative to the 2 yr after the transition) and at the last test (0.67 ± 1.33 mo) before the transition.

To determine individual producer ability to perceive actual change, thresholds were defined to evaluate when the actual performance difference was considered different from 0. A milk yield change between −417 and +929 kg/cow per year was considered to be not different from 0 and therefore no milk yield change following AMS introduction. These changes were used as thresholds for classifying whether producer perception aligned with reality. For the SCC change, −50,000 and +48,000 cells/mL were used as thresholds. The proportion of dairy producers who accurately perceived their milk yield and SCC changes after AMS introduction was 39.4% (increase: 36.3%; decrease: 100.0%; and no change: 45.5%) and 46.7% (increase: 50.0%; decrease: 39.0%; and no change: 54.1%), respectively. A total of 63.7 and 61.0% of dairy producers perceived an increase in milk yield and a decrease in SCC level, respectively, when it was not actually the case; these results indicate that some dairy producers either were not aware of the actual changes or distorted the changes when providing their response. It is possible that those who distorted their changes preferred to believe that AMS introduction enhanced performance because they invested a lot of money into the adoption of that system. This may be caused by a cognitive bias that leads the purchaser of an expensive product to see through the product's faults as a way to justify their purchase (Cohen and Goldberg, 1970).

Milk yield and SCC change averages, according to categories (increase, decrease, and no change) of dairy producer perception and reality, are depicted in Table 2. Regardless of producer perception, actual performance changes were similar within the same categories. Indeed, the actual milk yield increase averaged 1,591 and 1,393 kg/cow per year for dairy producers who perceived an increase and no change in milk yield after the transition, respectively. Half of the dairy producers who perceived that milk yield increased after AMS adoption were classified within the no change category, mainly because the increase was not substantial enough to be considered different from 0 according to our thresholds. Further analyses were performed to characterize herds in which producers accurately perceived the actual milk yield and SCC changes after AMS adoption and those that did not according to 3 performance indicators (herd milk yield average, SCC, and culling rate before and after AMS transition). Herds in which producers accurately perceived the actual milk yield increase after AMS transition were characterized by a greater average herd milk yield following AMS adoption (mean ± SE; 10,456 ± 205 vs. 9,213 ± 196 kg/cow per year; *P* < 0.001) compared with herds in which producers did not perceive the actual increase. In contrast, herds in which producers discerned the actual milk yield increase after AMS transition tended to have lower average milk yield before AMS adoption compared with herds in which producers did not perceive the actual increase (8,856 ± 201 vs. 9,279 ± 156 kg/cow per year; *P* = 0.10). Moreover, average herd milk yield after AMS adoption tended to be about 947 kg/cow per year greater in herds in which producers accurately perceived no change in actual milk yield and SCC after AMS transition than in herds in which producers did not perceive the stable performance (*P* ≤ 0.09). For dairy producers who accurately discerned their actual SCC decrease after AMS adoption, herd-average SCC was 69,000 cells/mL greater before the AMS adoption compared with herds in which producers did not perceive the actual decrease (*P* ≤ 0.02). No difference in culling rate was detected between herds in which producers were able or not able to perceive their actual changes (*P* > 0.13).

**Table 2:** Milk yield and SCC change (mean ± SD; minimum to maximum in parentheses) according to perception and reality categories for 97 herds that adopted an automated milking system^1^

Perception	Reality		
	Increase	Decrease	No change
Milk yield (kg/cow per year)			
Increase	1,591 ± 466 (946 to 2,605)	−1,041 ± 587 (−2,292 to −460)	201 ± 386 (−396 to 914)
	n = 29	n = 11	n = 40
Decrease	—	−984 ± 658 (−1,743 to −602)	—
		n = 3	
No change	1,393 ± 490 (1,107 to 1,959)	−1,286 ± 685 (−2,060 to −760)	119 ± 223 (−223 to 342)
	n = 3	n = 3	n = 5
SCC (×10^3^ cells/mL)			
Increase	106.6 ± 53.9 (59.0 to 202.0)	—	6.8 ± 30.2 (−37.0 to 37.0)
	n = 7		n = 7
Decrease	130.4 ± 88.1 (50.0 to 291.0)	−109.8 ± 49.2 (−242.0 to −62.0)	−6.9 ± 26.8 (−49.0 to 45.5)
	n = 6	n = 16	n = 19
No change	100.2 ± 44.2 (69.0 to 200.0)	−91.9 ± 32.4 (−147.5 to −55.0)	3.0 ± 23.0 (−47.0 to 42.0)
	n = 9	n = 8	n = 20

1 Valid data for both perception and reality were obtained for 94 and 92 herds for milk yield and SCC, respectively. Milk yield and SCC changes were calculated as differences between the 12-mo rolling herd-average milk yield and SCC at the closest milk test 2 yr after transitioning to AMS (mean ± SD; 0.66 ± 1.45 mo relative to the 2 yr after the transition) and at the last test (0.67 ± 1.33 mo) before the transition.

In summary, dairy producers were, on average, able to accurately perceive their increase or decrease in milk yield and SCC following AMS introduction. Nevertheless, the results indicate that some dairy producers either were not aware of their actual change or distorted their view of that change following AMS adoption, as 63.7 and 61.0% perceived a milk yield increase and an SCC decrease after the milking system change, respectively, whereas that was not the case in reality.
